# Utility of Synovial Alpha Defensin Lateral Flow Test in Diagnosing Septic Arthritis in Native Knees

**DOI:** 10.7759/cureus.112425

**Published:** 2026-07-10

**Authors:** Dang-Huy Do, Brandon Wood, Ishvinder S Grewal

**Affiliations:** 1 Department of Orthopedic Surgery, University of Texas Southwestern Medical Center, Dallas, USA

**Keywords:** alpha defensin, infection, knee, native, septic arthritis, synovial fluid

## Abstract

Introduction: Septic arthritis of native joints requires prompt diagnosis to prevent joint destruction and sepsis. Traditional diagnostic methods, such as synovial fluid culture and cell counts, are time-consuming and can be unreliable. The Synovasure® Alpha Defensin Lateral Flow Test (Zimmer Biomet, Claymont, DE, USA) detects alpha defensin in synovial fluid and is well-established for diagnosing periprosthetic joint infections. However, its diagnostic value in native joints remains insufficiently studied.

Methods: This prospective cohort study enrolled 25 adults presenting with suspected septic arthritis of the knee. Patients were excluded if they had prior knee arthroplasty or orthopedic implants. Demographics, comorbidities, vitals, and lab values were collected. Patients who tested positive for alpha defensin on aspirated synovial fluid were compared to those who tested negative. Infection was defined as a positive synovial culture, while gout was defined as positive synovial crystals. We calculated sensitivity, specificity, positive predictive value (PPV), and negative predictive value (NPV).

Results: Nineteen patients tested positive for alpha defensin, of whom four had culture-confirmed septic arthritis. All six alpha defensin test-negative patients had negative cultures. The test demonstrated a sensitivity of 100% (CI: 39%-100%), specificity of 29% (CI: 11%-52%), PPV of 21% (CI: 16%-25%), and NPV of 100% (CI: 54%-100%). Demographics, comorbidities, and serum inflammatory markers were similar between test-positive and test-negative groups. Alpha defensin test-positive patients had a significantly higher synovial polymorphonuclear neutrophil (PMN) percentage (p<0.001). All nine patients with positive synovial crystals tested positive (p=0.04).

Discussion: The high sensitivity and NPV of the Synovasure® Alpha Defensin Lateral Flow Test (Zimmer Biomet) demonstrate its potential utility to rapidly rule out septic arthritis in native knees. However, the test’s low specificity warrants caution, as false-positive results may occur with noninfectious inflammatory processes. While the rapid turnaround may aid in early triage and exclusion of infection, confirmatory evaluation with synovial culture remains essential to prevent misdiagnosis and overtreatment.

## Introduction

Infection leading to septic arthritis of native joints is a significant concern in emergency departments and hospitals. Early diagnosis of a septic joint in these settings is critical, as timely operative intervention can prevent sepsis and joint degeneration [1-3]. However, diagnosis of septic arthritis is often delayed as subsequent lab processing, cell count differential, Gram staining, and cultures following arthrocentesis may require hours to days to finalize. This processing time delays prompt care and can negatively impact patient recovery, resulting in disability and an elevated risk of mortality [1, 3]. Additionally, for fluid cell count results, cutoffs for infection are widely variable and range from 25,000 to 85,000 nucleated cells [4, 5]. Furthermore, these cutoff values can be confounded by immunosuppression from chemotherapy or an overactive inflammatory response to crystal arthropathy, such as gout or pseudogout [6].

Serum sedimentation rate (ESR), C-reactive protein (CRP), and synovial polymorphonuclear cells (PMNs) are commonly used markers in screening for a septic joint [7]. Alpha defensin is another marker present in the synovial fluid that has been the subject of various studies. This is an antimicrobial peptide released by neutrophils in response to bacterial pathogens [8]. Deirmengiean et al. found this biomarker to have 100% sensitivity and specificity in the diagnosis of periprosthetic joint infections [9]. Traditionally, testing for this was performed in the hospital laboratory or sent out externally. However, the recently developed Synovasure® Alpha Defensin Lateral Flow Test (Zimmer Biomet, Claymont, DE, USA) allows clinicians to quickly test for synovial fluid alpha defensin in less than five minutes. This test has been proven to be a reliable indicator for infection in periprosthetic joint infections and, despite only being applied within the last 10 years, it has become widely accepted [10, 11].

While the alpha defensin test has been studied extensively in periprosthetic joints, there is limited data and guidance on its usage in native joints. Prior studies have primarily evaluated a laboratory-based alpha defensin test or examined the pediatric population [12, 13]. The goals of this study are to evaluate the ability of the rapid synovial alpha defensin lateral flow test as a rule-out tool for infection in native adult knees.

## Materials and methods

Study design

This was a prospective cohort study of patients being evaluated for septic arthritis of a native knee joint at a tertiary referral center between April 2025 and April 2026. Institutional Review Board, University of Texas Southwestern Medical Center approval was obtained for this study (STU-2023-0091, dated December 2, 2025). Patients included in the study were at least 18 years old and all presented with clinical and physical exam findings consistent with septic arthritis of the knee, including knee joint pain, effusion of the knee, overlying erythema, and limited knee range of motion with loss of over 40 degrees of motion. There is no universally accepted standardized criterion for septic arthritis of the knee, so these common clinical and physical exam findings were used prior to obtaining laboratory tests. Additionally, patients were only included if there was an excess of at least 5 mL of fluid following aspiration, to maintain standard-of-care laboratory examination. Exclusion criteria included those with a previous knee replacement or orthopedic implants. Additionally, the alpha defensin test was not performed on patients with significant hemarthrosis upon aspiration due to a known potential false-negative result with the test, according to the manufacturer. There was one patient identified with a significant hemarthrosis for whom testing was not performed.

Data collection

Demographic data, including age, sex, race, and body mass index (BMI), were collected along with common comorbidities such as type II diabetes mellitus, hypertension, and tobacco usage. Objective clinical parameters, including maximum temperature, maximum heart rate, and maximum systolic blood pressure, were obtained for each patient. Laboratory values were recorded, including serum ESR, CRP, procalcitonin, white blood cell count (WBC), and synovial fluid studies (cell count with differential, culture, Gram stain, and crystal analysis). A positive bacterial culture was used as the reference standard for septic arthritis in our study, consistent with prior studies examining alpha defensin’s utility [12, 13].

Testing procedure

Following aspiration of the suspected infected joint and sending the required fluid to the lab according to standard clinical protocol, the remaining synovial fluid was tested with the Synovasure® Alpha Defensin Lateral Flow Test (Zimmer Biomet). The proper testing procedure, as determined by the manufacturer, Zimmer Biomet, was performed.

Statistical analysis

A pre-hoc power analysis was performed, indicating a minimum sample size of 22 patients was required for at least 80% power and a 5% significance level. In the power analysis calculation, derived from findings by Cooper et al., the primary end point was the specificity of alpha-defensin for native knee septic arthritis and was based on the sample size that assumed a specificity of 72%, a sensitivity of 100%, and a septic arthritis prevalence of 10% [12]. Two cohorts were compared based on the result of the alpha defensin test: alpha defensin-positive versus alpha defensin-negative. Mean and standard deviation were calculated for age, BMI, and continuous lab values, and unpaired T-tests were used to compare these variables between cohorts. Chi-squared tests were used to compare sex, the presence of comorbidities, culture results, and the presence of crystals in the synovial fluid, and a Fisher’s exact test was used to compare race. The sensitivity, specificity, negative predictive value (NPV), and positive predictive value (PPV) of the alpha defensin test were calculated with corresponding 95% confidence intervals. For this study, statistical significance was set at p<0.05.

## Results

A total of 25 patients were enrolled in the study. These patients ranged in age from 29 to 77 years with a mean age of 52.3 years. Seventy-two percent of the patient population were Hispanic, 16% were white, and 12% were African American. Regarding sex, 56% of the patients were male. When classified by alpha defensin assay result, there were no significant differences between the cohorts regarding demographics or comorbidities (Table 1).

**Table 1:** Patient characteristics

Variables	Alpha defensin-positive	Alpha defensin-negative	Test statistic	p-value
Age in years				
Mean (SD)	54.5 (14.5)	45.2 (5.5)	t = 1.52	0.14
Range	29 - 77	40 - 56		
Race				
White non-Hispanic, N (%)	3 (16%)	1 (17%)	n/a	0.80
African American, N (%)	3 (16%)	0 (0%)		
Hispanic, N (%)	13 (68%)	5 (83%)		
Male sex, N (%)	11 (58%)	3 (50%)	X^2 ^= 0.11	0.73
Comorbidity				
BMI (Mean [SD])	27.7 (3.7)	27.6 (4.6)	t = 0.05	0.96
Diabetes, N (%)	4 (21%)	2 (33%)	X^2 ^= 0.36	0.54
Hypertension, N (%)	12 (63%)	2 (33%)	X^2 ^= 1.58	0.20
Tobacco use, N (%)	7 (37%)	1 (17%)	X^2 ^= 0.82	0.36

Of the 25 patients included in the study, 19 (76%) tested positive using the Synovasure® Alpha Defensin Lateral Flow Test (Zimmer Biomet). These patients had a significantly higher percentage of polymorphonuclear cells (PMNs) in the synovial fluid when compared to patients with a negative test (91.2% vs. 46.5%, p<0.001, Table 2). Additionally, this cohort had a higher percentage of crystals present in the synovial fluid (47% vs. 0%, p=0.04). There were no differences between the cohorts with regard to vitals or other inflammatory lab markers.

**Table 2:** Patient inflammatory markers and lab data *p-value <0.05 ESR: erythrocyte sedimentation rate; CRP: C-reactive protein; PMN: polymorphonuclear neutrophil

Variables	Alpha defensin-positive	Alpha defensin-negative	Test statistic	p-value
Maximum temperature (°C)				
Mean (SD)	37.4 (0.7)	37.3 (0.7)	t = 0.16	0.87
Range	36.2 - 39.1	36.4 - 38.4		
Maximum heart rate (beats per minute)				
Mean (SD)	101.7 (13.2)	90.2 (22.5)	t = 1.57	0.13
Range	76 - 124	59 - 122		
Maximum systolic blood pressure (mmHg)				
Mean (SD)	144 (20)	141.3 (21)	t = 0.05	0.95
Range	101 - 190	116 - 171		
ESR (mm/hr)				
Mean (SD)	40.4 (26.3)	20.5 (10.0)	t = 1.46	0.16
Range	3 - 88	7 -31		
CRP (mg/dL)				
Mean (SD)	14.2 (8.4)	10 (11.7)	t = 0.98	0.34
Range	2.4 - 29.9	0.7 - 32		
Serum procalcitonin				
Mean (SD)	0.63 (0.89)	0.03 (0.02)	t = 1.13	0.29
Range	0.06 - 2.75	0.02 - 0.05		
Serum WBC (cells/μL)				
Mean (SD)	11.2 (4.2)	12.1 (5.0)	t = 0.45	0.66
Range	6.6 - 21.8	5.91 - 18.3		
Synovial nucleated cells				
Mean (SD)	54,904 (40,865)	19,856 (41,046)	t = 1.83	0.08
Range	2,797 - 157,000	153 - 103,284		
Synovial PMN (%)				
Mean (SD)	91.2 (4.7)	46.5 (28.9)	t = 6.78	<0.001*
Range	82 - 99	8 - 85		
Crystals present?				
Yes, N (%)	9 (47%)	0 (0%)	X^2 ^= 4.26	0.04*
No, N (%)	10 (53%)	6 (100%)		
Culture results				
Culture positive, N (%)	4 (21%)	0 (0%)	X^2 ^= 1.44	0.22
Culture negative, N (%)	15 (79%)	6 (100%)		

There were four culture-positive samples. These samples were all alpha defensin-positive as well, representing 21% of the 19 patients in the alpha defensin-positive cohort. These patients tested positive for a variety of bacteria, including *Enterobacter cloacae* complex, *Pseudomonas aeruginosa*, *Staphylococcus aureus*, and bacteria from the *Streptococcus mitis/oralis *group. Anaerobic, fungal, and acid-fast bacillus cultures were found to be negative in all 19 samples.

The alpha defensin-positive group included nine (47%) patients with crystals present in the synovial fluid, while none of the alpha defensin-negative patients had crystals present. Of the crystal-positive patients, seven were found to be Gram stain- and culture-negative. Six of the nine crystal-positive patients were found to have calcium pyrophosphate crystals, while the remaining three patients presented with uric acid crystals.

For native knee infection, we found the sensitivity of the Synovasure® Alpha Defensin Lateral Flow Test (Zimmer Biomet) to be 100% (CI: 39%-100%), and the specificity of the test was 29% (CI: 11%-52%). The PPV was 21% (CI: 16%-25%), and the NPV was 100% (CI: 54%-100%).

Among 15 patients with false-positive results (alpha defensin-positive and culture-negative), nine (60%) had crystalline arthropathy, and two (13%) had inflammatory arthropathy. Stratified results demonstrating the exclusion of patients with crystalline arthropathy, inflammatory arthropathy, or both from our cohort are demonstrated in Table 3.

**Table 3:** Stratified cohort excluding crystalline and/or inflammatory arthropathy PPV: positive predictive value; NPV: negative predictive value

Stratified cohort	Sensitivity	Specificity	PPV	NPV
Overall cohort	100%	29%	21%	100%
Excluding crystalline arthropathy (n=9)	100%	50%	40%	100%
Excluding rheumatoid arthritis (n=2)	100%	31%	23%	100%
Excluding both crystalline arthropathy and rheumatoid arthritis (n=11)	100%	60%	50%	100%

## Discussion

In this prospective cohort of patients presenting with suspected septic arthritis of the native knee, the Synovasure® Alpha Defensin Lateral Flow Test (Zimmer Biomet) demonstrated excellent sensitivity and NPV, though it is important to note the wide confidence intervals for both of these. In contrast, specificity and PPV were markedly lower, indicating that it is not reliable for confirming septic arthritis. These performance characteristics were driven by a high rate of false-positive tests, as 71% of culture-negative patients tested positive on the assay, while no culture-positive patients tested negative. The elevated false-positive rate was at least partially attributable to crystalline arthropathy, which was identified in nine of the alpha defensin-positive patients, seven of whom were culture-negative.

These findings closely align with the limited existing literature evaluating alpha defensin testing in native joints, which has reported high sensitivity but variable specificity. Cooper et al. used a laboratory alpha defensin test and observed similar performance metrics of the test with 100% sensitivity and NPV, with low specificity and PPV due to a 28% false-positive rate [12]. Machida et al. performed a similar study on pediatric patients and also had a 0% false-negative rate, but did not report specificity and sensitivity [13]. Although these studies differed from the present investigation in the type of alpha defensin assay employed, patient population, or joints tested, the overall pattern of results is comparable and supports the utility of the alpha defensin lateral flow test as a reliable tool for excluding septic arthritis in native knees.

The high sensitivity and NPV observed in this study likely stem from the alpha defensin test’s ability to detect inflammation in the synovial fluid, irrespective of its underlying etiology. Alpha defensins are antimicrobial peptides released by neutrophils as part of the innate immune response and exhibit activity against a broad range of pathogens [8, 14]. Consequently, a negative test result indicates that the level of neutrophilic inflammation in the joint is below the detection threshold of the assay. This accounts for the complete absence of false-negative results in the present cohort and underscores the test’s value as a rapid rule-out tool for septic arthritis in native knees.

The relatively low specificity observed in this study is explained by the same biological mechanism that confers the test’s high sensitivity. Although the peptide is triggered by bacterial pathogens, it is also elaborated in non-infectious inflammatory conditions such as gout, pseudogout, or autoimmune arthropathies [15]. There were 15 false positives, or those who were alpha defensin-positive but culture-negative. Of these, 60% were due to crystalline arthropathy, and 13% were due to inflammatory arthropathy, specifically rheumatoid arthritis previously diagnosed by a rheumatologist. Notably, no patients in the alpha defensin-negative group or in the culture-positive groups had documented inflammatory arthropathy or crystals. After excluding patients with crystalline arthropathy and rheumatoid arthritis, specificity improved from 29% to 60%, while PPV improved from 21% to 50%. NPV and sensitivity remained unchanged. This pattern reflects the test’s responsiveness to synovial inflammation regardless of etiology, which is critical to understanding potential false positives with the test. This observation is corroborated by the significantly higher synovial PMN percentage in the alpha defensin-positive group. Consequently, non-infectious inflammation confounds the assay result and limits its utility for confirming a diagnosis of septic arthritis.

The Synovasure® Alpha Defensin Lateral Flow Test (Zimmer Biomet) demonstrated low specificity (29%) and positive predictive value (21%), rendering it unreliable for confirming a diagnosis of septic arthritis in native knees-a setting in which its performance differs from that observed in periprosthetic joint infections. Nevertheless, its 100% sensitivity and negative predictive value establish it as a valuable rapid rule-out tool. This distinction is especially relevant in the emergency department, where clinical findings of septic arthritis frequently overlap with more common non-infectious conditions. Carpenter et al. reported that only 7% of patients who underwent arthrocentesis for suspected septic arthritis in the ED ultimately had culture-positive results [16]. Despite this low prevalence, septic arthritis requires time-sensitive antibiotics and surgical intervention when present [17]. Consequently, this test enables emergency department physicians to rapidly narrow the differential diagnosis and potentially avoid unnecessary interventions.

There are some limitations to this study. Our sample size was relatively small due to the availability of testing kits. Nonetheless, a pre-hoc power analysis was performed to ensure our sample size was sufficiently powered, and other studies evaluating alpha defensin for native joints had similar sample sizes [12, 13]. This, along with the fact that this study was performed at a single center, may limit the generalizability of our findings to other institutions and populations. The small cohort size also lent itself to a small number of culture-positive cases. It is important to note that “culture-negative but clinically treated” septic arthritis can still occur. Because our study only uses positive bacterial cultures as the standard for having septic arthritis, this may misclassify culture-negative infection as aseptic disease. So, alpha defensin-positive culture-negative septic arthritis may be counted as false positives, which leads to underestimation of the test’s specificity and PPV for all forms of septic arthritis. However, there is no universal criterion or test to absolutely diagnose all septic joints. Correspondingly, culture positivity is the most commonly used and consistent marker for diagnosing septic arthritis and is consistent with other studies evaluating alpha defensin tests [12, 13]. Additionally, in order not to disrupt the standard of care for patients evaluated in the emergency room with a suspicion of septic arthritis, we only performed the test on patients who had at least 5 mL of synovial fluid for study testing. This may introduce some selection bias by excluding patients with smaller joint effusions. Finally, there was one patient who had a hemarthrosis upon aspiration, for whom alpha-testing was not performed due to the manufacturer’s recommendation of a high false-negative rate. This may introduce additional selection bias and reduce generalizability by excluding patients with bloody joint effusions.

## Conclusions

The Synovasure® Alpha Defensin Lateral Flow Test (Zimmer Biomet) demonstrated excellent sensitivity and negative predictive value in this preliminary prospective cohort study, suggesting it has the potential to be a reliable, rapid rule-out tool for septic arthritis in native knees. These performance characteristics have the potential to allow physicians to quickly exclude infection in patients with nonspecific symptoms and narrow the differential diagnosis. However, the wide confidence intervals for sensitivity and negative predictive value, along with low specificity and positive predictive value, driven by frequent false positives in the setting of crystal arthropathy and sterile neutrophilic inflammation, suggest that the test is less suitable for confirming septic arthritis and must be interpreted within the full clinical and laboratory context. Future larger multicenter studies are warranted to further validate the diagnostic utility of the rapid alpha defensin lateral flow test.
